# Deep learning-based ultrasonographic classification of canine chronic kidney disease

**DOI:** 10.3389/fvets.2024.1443234

**Published:** 2024-09-04

**Authors:** Heejung Yu, In-Gyu Lee, Jun-Young Oh, Jaehwan Kim, Ji-Hoon Jeong, Kidong Eom

**Affiliations:** ^1^Department of Veterinary Medical Imaging, College of Veterinary Medicine, Konkuk University, Seoul, Republic of Korea; ^2^Department of Computer Science, College of Electrical and Computer Engineering, Chungbuk National University, Cheongju-si, Republic of Korea

**Keywords:** chronic kidney disease, canine, artificial intelligence, deep learning-based disease diagnosis, ultrasonographic classification

## Abstract

**Objectives:**

In veterinary medicine, attempts to apply artificial intelligence (AI) to ultrasonography have rarely been reported, and few studies have investigated the value of AI in ultrasonographic diagnosis. This study aimed to develop a deep learning-based model for classifying the status of canine chronic kidney disease (CKD) using renal ultrasonographic images and assess its diagnostic performance in comparison with that of veterinary imaging specialists, thereby verifying its clinical utility.

**Materials and methods:**

In this study, 883 ultrasonograms were obtained from 198 dogs, including those diagnosed with CKD according to the International Renal Interest Society (IRIS) guidelines and healthy dogs. After preprocessing and labeling each image with its corresponding IRIS stage, the renal regions were extracted and classified based on the IRIS stage using the convolutional neural network-based object detection algorithm You Only Look Once. The training scenarios consisted of multi-class classification, categorization of images into IRIS stages, and four binary classifications based on specific IRIS stages. To prevent model overfitting, we balanced the dataset, implemented early stopping, used lightweight models, and applied dropout techniques. Model performance was assessed using accuracy, recall, precision, F1 score, and receiver operating characteristic curve and compared with the diagnostic accuracy of four specialists. Inter- and intra-observer variabilities among specialists were also evaluated.

**Results:**

The developed model exhibited a low accuracy of 0.46 in multi-class classification. However, a significant performance improvement was observed in binary classifications, with the model designed to distinguish stage 3 or higher showing the highest accuracy of 0.85. In this classification, recall, precision, and F1 score values were all 0.85, and the area under the curve was 0.89. Compared with radiologists, whose accuracy ranged from 0.48 to 0.62 in this experimental scenario, the AI model exhibited superiority. Intra-observer reliability among radiologists was substantial, whereas inter-observer variability showed a moderate level of agreement.

**Conclusions:**

This study developed a deep-learning framework capable of reliably classifying CKD IRIS stages 3 and 4 in dogs using ultrasonograms. The developed framework demonstrated higher accuracy than veterinary imaging specialists and provided more objective and consistent interpretations. Therefore, deep-learning-based ultrasound diagnostics are potentially valuable tools for diagnosing CKD in dogs.

## 1 Introduction

Chronic kidney disease (CKD) is the most prevalent renal condition in elderly dogs, with an occurrence rate of ~0.5%−1.5% in the overall population (1). It is characterized by lasting structural or functional impairment of the kidneys that persists for at least 3 months (2). CKD can develop due to various factors, such as age-related degeneration, genetic predisposition, infections, toxins, and immune-mediated conditions, presenting with non-specific symptoms, such as weight loss, vomiting, and lethargy (3). The diagnosis and staging of CKD follow the guidelines set forth by the International Renal Interest Society (IRIS). Various methods, including hematological and imaging tools, have been used to diagnose CKD. Ultrasonography is a non-invasive and cost-effective real-time method that plays a key role in diagnostic imaging.

Canine CKD exhibits several sonographic features, including irregular contours, increased echogenicity, decreased corticomedullary differentiation, and decreased renal volume (4). Moreover, renal function is associated with renal cortical thickness (5) and these parameters correlate with CKD severity (6). However, despite these imaging clues, predicting the degree of renal function impairment through ultrasonography is challenging. The interpretation of ultrasonograms is subjective, with inconsistencies in diagnostic criteria. Additionally, the accuracy varies depending on the radiologist's experience.

Artificial intelligence (AI) is a comprehensive field of study that involves creating computer systems to mimic human intelligence, encompassing problem-solving, learning, and pattern recognition. Deep learning is a field of AI that involves constructing artificial neural networks, which are similar in structure to the human brain, to learn from data. This allows machines to think and make decisions like humans (7). It is actively used in various industries such as manufacturing, aviation, aerospace, and electronics, and is also being extensively researched in the medical field (8, 9). For example, the U-Net deep learning convolutional neural network (CNN) based model is primarily used for image segmentation the biomedical field. It is employed in tasks such as vascular image segmentation and identifying cells or other pathological features in tissue samples (10). In human medicine, AI represents a pioneering and efficient approach for the ultrasonographic diagnosis of CKD, with numerous AI-based ultrasound studies on CKD having been published (11). These encompass research utilizing traditional machine learning-based radiomics, as well as deep learning studies employing CNN-based architectures (12, 13). Some of these studies have compared AI models with radiologists (14).

In veterinary medicine, various studies applying AI to imaging diagnosis have been recently published. These studies have predominantly been conducted in radiographic modalities (15–17), and there have been recent cases of AI application in other advanced modalities like MRI as well (18). Regarding the kidney diseases, there are still relatively few studies applying AI across all modalities. A study on automatically measuring kidney volume from CT scans was published in 2022 (19), and a study on renal calculi detection was reported in 2023 (20). To the best of the author's knowledge, there have been no studies applying AI to kidney ultrasound.

The purposes of this study were to develop a deep learning model for classifying CKD stages in dogs based on renal ultrasonograms and to compare its accuracy with that of radiologists. The goal was to explore the potential of new diagnostic approaches for enhancing the accuracy and objectivity of medical image evaluation.

## 2 Materials and methods

### 2.1 Ethics statements

Written informed consent was obtained from the canines' owners for their study participation.

### 2.2 Study population and database construction

This study included dogs that visited the Veterinary Medical Teaching Hospital, Konkuk University, between November 2015 and November 2023. To select the study population, electronic medical charts were filtered using the keyword “IRIS stage,” and the medical records of these patients were reviewed. As a group of patients with CKD, we included those diagnosed and classified according to the CKD IRIS stage, with both blood tests and ultrasound examinations conducted simultaneously or within a maximum interval of 4 weeks. Patients who showed no abnormalities in either renal function parameters or ultrasound findings during the same study period were included in the control group, which was named “IRIS stage 0” for convenience. The collected clinical data included age, sex, breed, blood symmetric dimethyl arginine (SDMA) concentration, creatinine level, blood urea nitrogen (BUN) level, and urine protein creatinine ratio (UPC). The ultrasonographic examination of each patient was reviewed, and sagittal plane images were obtained as much as possible from the optimal frame of the video on both sides and bilateral still shots of the kidneys. Cases where the kidneys were not clearly visualized on ultrasonography were excluded from the analysis. All the images were acquired from two different machines (Prosound F75^®^ Hitachi-Aloka Medical, Ltd., Tokyo, Japan, and Canon Apolio i800^®^ Toshiba Medical, Tokyo, Japan), evaluated using a picture archiving and communication system (INFINITT PACS; INFINITT Healthcare, Korea), and saved in PNG file format. Imaging reports were also documented as the ratio of the renal length to the aortic diameter. In total, 883 renal images were obtained from 198 dogs.

### 2.3 Data preprocessing

Before training, the data was preprocessed to make them suitable for the learning process. Initially, the image sizes were standardized. Given that the images varied in size, a center-cropping approach was employed to resize them, ensuring ease of application of the model. Subsequently, image-processing techniques were applied to eliminate annotations drawn by the veterinarians during the examination. An image processing library called “OpenCV” was used for this task. Then, the RGB images were transformed into the HSV color space, and a binary mask was created by extracting green tones from the images. This mask was used to remove any remaining annotations. Next, to further enhance the image quality, a Laplacian filter was used to sharpen the images make the information on the ultrasound image clearer. These preprocessing steps aimed to create a uniform dataset free of unnecessary annotations and with enhanced image clarity, making it well-suited for training machine learning models in the context of veterinary diagnoses.

**Figure 1:**
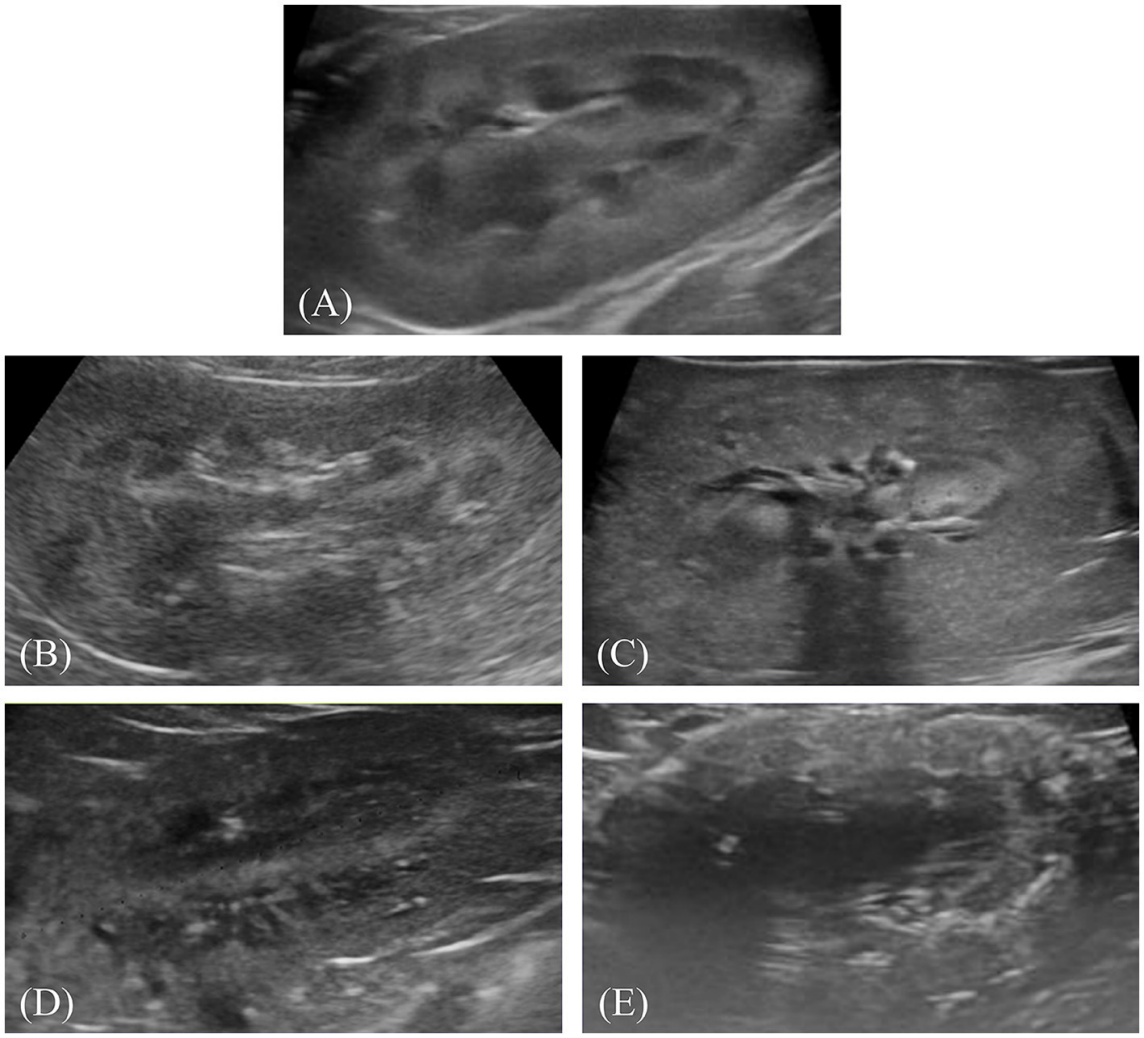
Representative ultrasonograms of the kidney by stages of chronic kidney disease according to the International Renal Interest Society. Each image is labeled as follows: **(A)** stage 0, **(B)** stage 1, **(C)** stage 2, **(D)** stage 3, and **(E)** stage 4.

After preprocessing, the images were labeled for classification. Labeling involved physically drawing bounding boxes around selected objects and assigning class names to these annotations as stored metadata for a class file. This was performed using the cloud-based software “Roboflow (Roboflow Inc, USA)”. An experienced veterinary radiologist (HY) annotated the bounding boxes within the ultrasonograms to fit around the kidney border and labeled the IRIS stage. Labeling was assigned in five stages, ranging from 0 to 4 (Figures 1A–E).

### 2.4 Developing convolutional neural network models for predicting chronic kidney disease status

You Only Look Once version 8-n (YOLO-v8-n), a convolutional network-based object detection model, was employed in the experimental setup. YOLO-v8-n is a lighter model with fewer layers than other models, which helps prevent overfitting by preventing the model from becoming too tuned to the training data. This implies that the model learns simpler patterns, trains faster, and operates more efficiently than other models. In the model, the input image is first divided into an S x S grid of cells, and each cell predicts multiple bounding boxes. Each box contains information such as a box confidence score, indicating the likelihood of containing an object, and a class confidence score, indicating the accuracy of the predicted class. Subsequently, to refine predictions, a loss function is computed to retain only the box closest to the ground truth among multiple predicted boxes. The model iterates this process during training to adjust the weights of the network, optimizing them. Once training concludes, using the optimized weights, predictions of bounding boxes are made on unseen data for evaluation (21).

The experimental scenarios are divided into two main types: multi-class classification and binary classification. First, the multi-class classification scenario involves categorizing stages 0 to 4, predicting the actual IRIS stage of each renal image. When an image is input, the AI's response can be one of five stages (0–4). Next, the binary classification scenario involves dividing IRIS stages 0 to 4 into two groups and predicting whether each image belongs to the lower or higher group. For example, it predicts whether a renal image is below or above IRIS stage 3 (binary). In this case, the AI's response indicates the higher or lower group rather than a specific IRIS stage. There are four possible binary classifications: Stage 0 vs. 1–4, Stage 0–1 vs. 2–4, Stage 0–2 vs. 3–4, and Stage 0–3 vs. 4. We conducted training and experiments for all 4 cases.

The preprocessed data were divided into training, validation, and test sets at a ratio of ~7:1:1 for each experimental setting. Owing to the small amount of data, the proportions of the validation and test sets were minimized. To prevent the model from overfitting the training data, an analysis of the learning curve was conducted. Early stopping was conservatively set to 30, and the dropout technique was applied at a rate of 30% to mitigate overfitting.

**Figure 2:**
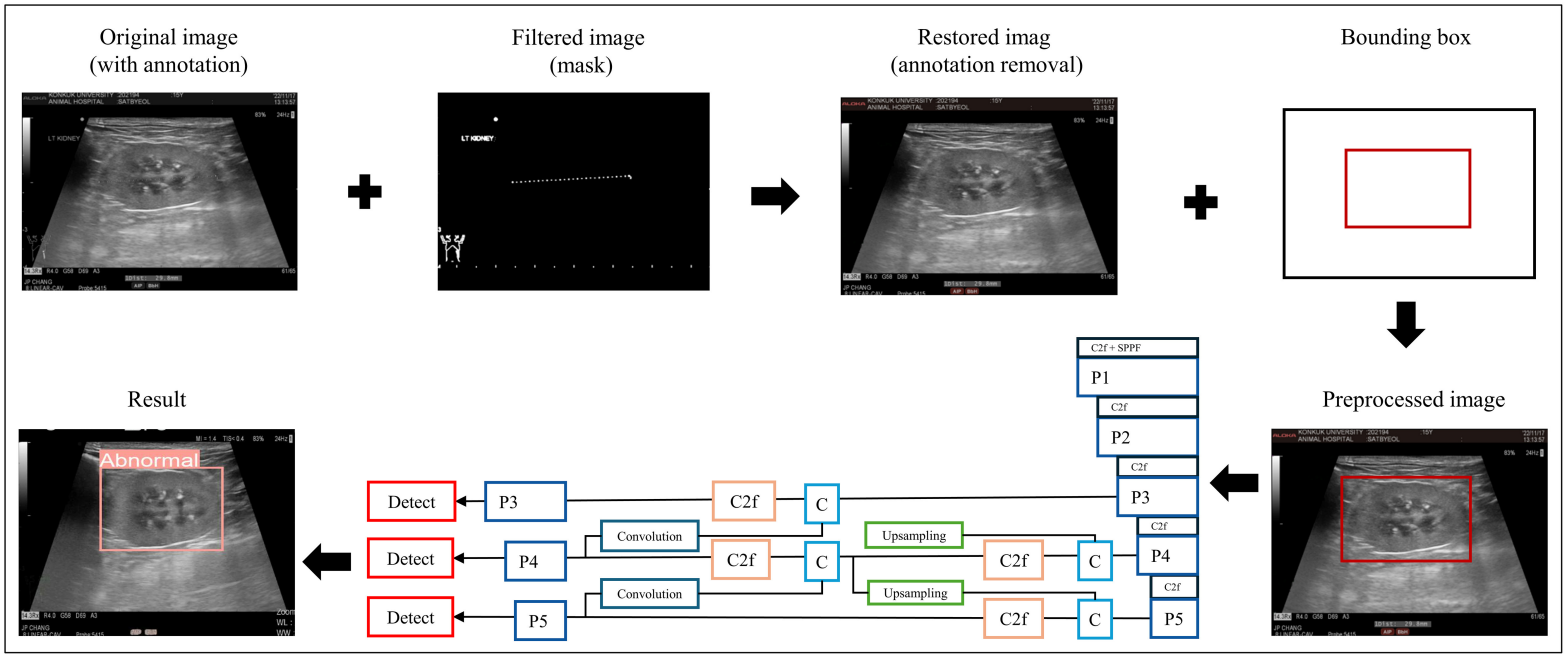
Data preprocessing and overall workflow. P, pooling layer; SPPF, spatial pyramid pooling fusion; C, Concat; U, upsample; C2f, faster implementation of the C2 module (CSP bottleneck with convolutions); CIoU, class intersection over union; DFL, distribution focal loss; BCE, binary cross-entropy loss.

The data-processing procedure and CNN architecture of the proposed model are shown in Figure 2.

### 2.5 Evaluation of model performance

The performance of the models during the training process was analyzed using training loss and validation loss curves. A confusion matrix was used to evaluate the performance of the classification model. The confusion matrix included true positive, false positive, true negative, and false negative, which were then used to calculate the accuracy, precision, recall (sensitivity), and F1 score, which served as performance metrics. These values were calculated as follows (22–25):


$$\begin{array}{r} Accuracy\;=\;\frac{TP+TN}{TP+TN+FP+FN}\\ Recall\;=\;\frac{TP}{TP+FN}\\ Precision\;=\;\frac{TP}{TP+FP}\\ F1\;score\;=\;2\times \frac{Precision\times Recall}{Precision+Recall} \end{array}$$

True Positive (TP): Instances correctly classified as positive by the model.

True Negative (TN): Instances correctly classified as negative by the model.

False Positive (FP): Instances incorrectly classified as positive by the model when they were actually negative.

False Negative (FN): Instances incorrectly classified as negative by the model when they were actually positive.

The receiver operating characteristic (ROC) curve and the area under the curve (AUC) were used for performance evaluation (23, 25). The AUC ranged from 0 (indicating that all classes were diagnosed incorrectly by the classifier) to 1 (reflecting perfect diagnostic performance across classes). These evaluation procedures were conducted across five experimental scenarios, as follows: one for multi-class classification and four for binary class classification.

### 2.6 Comparison with radiologists

Four radiologists performed the same diagnostic task using the test set to compare the performance of the developed model with that of the radiologist. This comparison was conducted only in the scenario where the model demonstrated its highest performance, distinguishing between stages 0–2 and 3–4 using stage 3 as the threshold. Radiologists were required to determine immediately whether renal images were above or below stage 3. This approach reflects the typical process in clinical practice, where radiologists' judgments are influenced by their accumulated experience and are highly subjective. This process was repeated twice with a one-week interval. The diagnostic performance of each radiologist was evaluated using the same metrics as those used for the developed model.

The inter- and intra-observer agreements of the radiologists were calculated. Inter-observer agreement was assessed using observations from each observer during the initial evaluation, whereas intra-observer reliability was determined by comparing each observer's observations between the initial and subsequent evaluations.

### 2.7 Statistical analysis

The study population and clinical data were subjected to normality test using the Shapiro–Wilk test. All continuous data are presented as the median and interquartile range (IQR). Spearman's correlation analysis was used to analyze the correlation between the IRIS stage (independent variable) and clinical data (dependent variables) including LK/AO, RK/AO, SDMA, BUN and Creatinine. The level of correlation was classified using the standard recommendation (26). Fleiss' and Cohen's kappa (κ) coefficients of agreement were used to evaluate the inter-observer agreement and intra-observer reliability of radiologists, respectively. The interpretation of κ was determined based on the Landis and Koch criteria: almost perfect (κ ≥ 0.80), substantial (0.60 ≤ κ < 0.80), moderate (0.40 ≤ κ < 0.60), fair (0.20 ≤ κ < 0.40), and poor (κ < 0.20) (27). All statistical significance was set at p < 0.05, and statistical analyses were performed using R statistical software (version 4.3.2; R Foundation, Vienna, Austria).

## 3 Results

### 3.1 Demographics and clinical data

In total, 198 dogs were included in this study, comprising 39 in IRIS stage 0, 44 in stage 1, 51 in stage 2, 35 in stage 3, and 29 in stage 4. The median age of all dogs was 11 years (IQR, 8.00–14.00 years), and the sex distribution was as follows: seven intact males (3.53%), 104 castrated males (52.52%), 7 intact females (3.53%), and 80 spayed females (40.4%). The median values for the left and right kidney length to aorta diameter ratio (LK/AO and RK/AO), SDMA, creatinine, and BUN of all the dogs were 6.7 (IQR, 5.9–7.5), 7.1 (IQR, 6.1–7.7), 20.0 (IQR, 13.0–36.2), 1.2 (IQR 0.8–2.0), and 28.0 (17.2–51.0), respectively. The UPC was measured in 122 dogs, with “over” observed in 8 cases, “under” observed in 19 cases, and a median measurement of 1.0 (IQR, 0.3–2.4) for the rest. The median ages of each group were 4.0 years (IQR, 2.5–6.0 years) for stage 0, 11.0 years (IQR, 11.0–13.0 years) for stage 1, 13.0 years (IQR, 5.9–7.2 years) for stage 2, 13.0 years (8.5–14.0 years) for stage 3, and 14.0 years (11.0–16.0 years) for stage 4 (Supplementary Table 1). The kidney length ratios (LK/AO and RK/AO) did not significantly change with IRIS stages (*p* > 0.05). However, SDMA (ρ = 0.913, *p* < 0.001), creatinine (ρ = 0.764, p < 0.001), and BUN (ρ = 0.699, *p* < 0.001) exhibited strong positive correlations with IRIS stages. These findings suggest that SDMA, creatinine, and BUN levels are closely associated with changes in IRIS stages.

### 3.2 Performance of the classification model

In multi-class and binary classification, the training loss decreased as the number of epochs increased, indicating that the models fit the training data well. When validated using the validation set, in all cases, as the number of epochs increased, the validation loss converged to approximately 0.6, with the minimum validation values ranging between 0.53 and 0.64. The training progressed well as the validation loss decreased consistently.

In multi-class classification, our model exhibited low performance, with an accuracy of 0.46. The F1 score, precision, and recall values were 0.46, 0.50, and 0.46, respectively. Attempting binary classification with specific stages set as thresholds exhibited a noticeable improvement in model performance. Across binary classification cases 1–4, improvements were observed in all performance metrics. Among these, case 3, which distinguished between CKD IRIS stages 0–2 and 3–4, exhibited the clearest classification. The performance achieved an accuracy, F1 score, precision, and recall of 0.85. The performance of the developed classification models is presented in Table 1. The confusion matrices for the classification models are shown in Supplementary Figure 1.

**Table 1:** Performance of the developed model in classifying the chronic kidney disease status of dogs: multi-class classification and binary classification.

**Classification**	**Accuracy**	**F1 score**	**Precision**	**Recall**
Multi-class	0.46	0.46	0.50	0.46
**Binary**				
Case 1^a^	0.65	0.72	0.70	0.65
Case 2^b^	0.78	0.79	0.78	0.76
Case 3^c^	0.85	0.85	0.85	0.85
Case 4^d^	0.80	0.76	0.83	0.80

^a^Experimental scenario for classifying chronic kidney disease International Renal Interest Society stages 0 and 1–4.

^b^Experimental scenario for classifying chronic kidney disease International Renal Interest Society stages 0–1 and 2–4.

^c^Experimental scenario for classifying chronic kidney disease International Renal Interest Society stages 0–2 and 3–4.

^d^Experimental scenario for classifying chronic kidney disease International Renal Interest Society stages 0–3 and 4.

**Figure 3:**
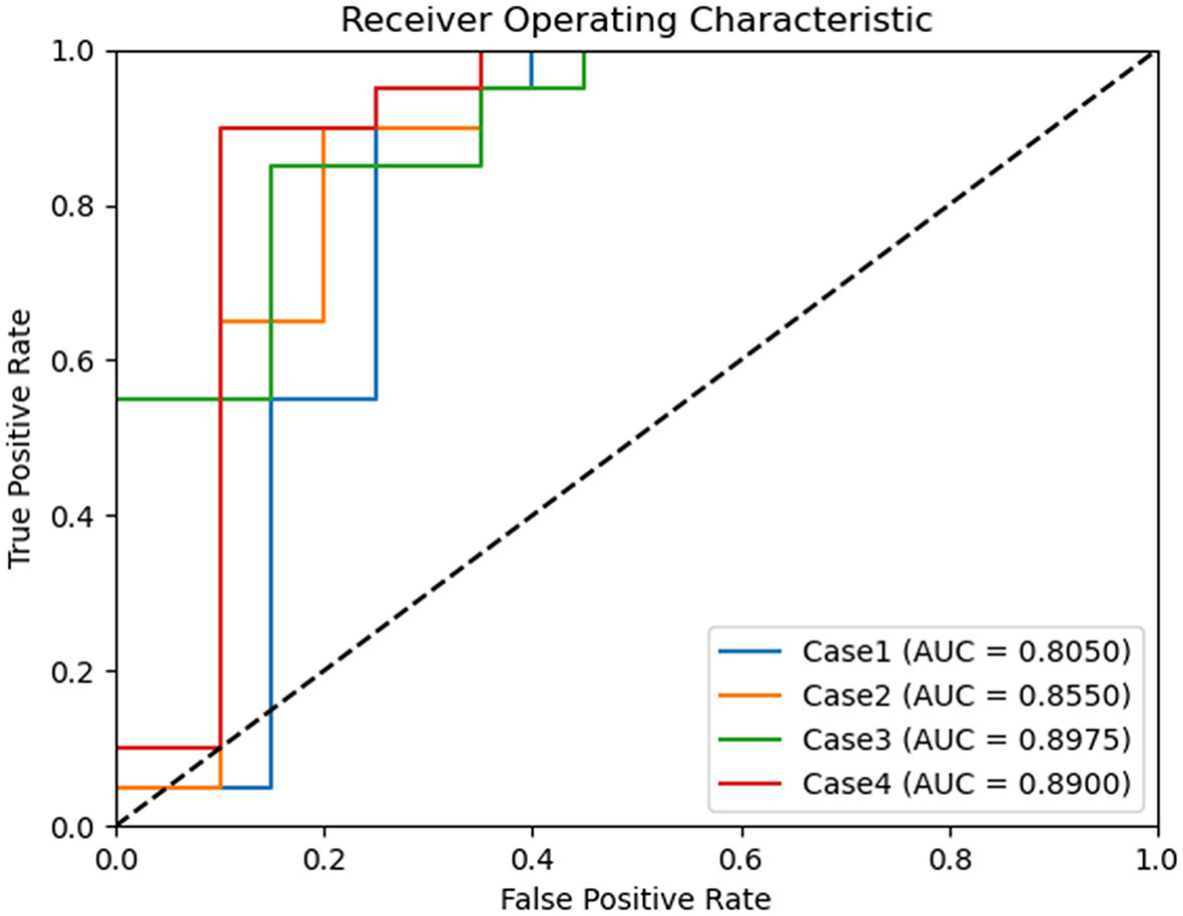
Comparison of receiver operating characteristic curves of binary classification models for chronic kidney disease status classification. Case 1 of binary classification distinguishes between chronic kidney disease International Renal Interest Society (IRIS) stages 0 and 1–4, case 2 between stages 0–1 and 2–4, case 3 between stages 0–2 and 3–4, and case 4 between stages 0–3 and 4. Among these, binary classification case 3 achieved the highest area under the curve, with a value of 0.897.

ROC curves were generated for each experimental scenario (cases 1–4) using binary classification. Notably, the best ROC curve with an AUC of 0.8975 was observed in case 3 (Figure 3).

### 3.3 Comparison between the deep learning model and radiologists

The performance of the four radiologists was evaluated for the binary classification of case 3, in which the developed model demonstrated the highest performance. The results confirmed the superior performance of the model compared with that of all the radiologists. The radiologist who demonstrated the best results achieved accuracy, F1 score, precision, and recall values of 0.62, 0.70, 0.70, and 0.70, respectively, which were lower than those of the model across all metrics. The accuracies of the remaining three radiologists were 0.48, 0.48, and 0.57, respectively (Table 2).

**Table 2:** Performance comparison between the deep learning model and radiologists in binary classification case 3^a^ of chronic kidney disease.

	**Accuracy**	**F1 score**	**Precision**	**Recall**
Deep learning model	0.85	0.85	0.85	0.85
**Radiologist**				
1	0.62	0.70	0.70	0.70
2	0.57	0.65	0.70	0.60
3	0.48	0.54	0.63	0.47
4	0.48	0.53	0.60	0.47
Average	0.54	0.60	0.66	0.56

^a^Experimental scenario for classifying chronic kidney disease International Renal Interest Society stages 0–2 and 3–4.

### 3.4 Inter-observer variability and intra-observer reliability of radiologists

Inter-observer agreement among the four radiologists was assessed based on their initial assessments. The obtained kappa value was 0.602, with a *p* < 0.001, indicating a statistically significant moderate level of agreement among the radiologists (Supplementary Table 2). Additionally, the intra-observer reliability was measured based on the first and second assessments by each radiologist. A significant agreement was observed between the first and second readings of all four radiologists. Two radiologists (3 and 4) demonstrated almost perfect agreement with kappa values of 0.879 and 0.855, respectively. The remaining two radiologists (1 and 2) exhibited substantial agreement with kappa values of 0.797 and 0.667, respectively. The calculated *p*-value for all four radiologists was < 0.001 (Supplementary Table 3).

## 4 Discussion

This study developed a deep learning framework for classifying ultrasonograms based on the CKD IRIS stages. Although the five-stage classification showed poor performance, a significant improvement was observed when simplified into a binary classification. The top-performing model, designed to distinguish CKD IRIS stage 3 or higher, exhibited consistently high levels of accuracy, precision, recall, and F1 score. This study was designed without data imbalance, indicating that our developed deep learning framework effectively predicts both positive (IRIS stage 3 or higher) and negative (IRIS stage < 3) classes. The model achieved accuracy comparable to that of a deep learning model reported in a similar study conducted in 2019 for distinguishing CKD IRIS stage 3 or higher from kidney images. In that study, a model was developed to determine whether an estimated glomerular filtration rate (eGFR) < 60 mL/min/1.73 m^2^ based on 4,505 ultrasonograms from 1,299 patients with CKD. The model achieved high-performance metrics with an accuracy of 0.85, AUC of 0.90, sensitivity of 0.61, and specificity of 0.92 (14). Kidney function is particularly susceptible to irreversible decline once eGFR decreases below 60 ml/min/1.73 m^2^, making this classification clinically significant (28). If similar criteria are established in veterinary medicine, our model is expected to have even greater clinical importance.

Applying a threshold between IRIS stages 2 and 3 yielded the best classification performance. However, explaining this result is difficult because deep learning models learn patterns from data without explicit programming, which obscures the optimal features used by the algorithm (7). Interestingly, several previous studies have reported significant imaging and clinical changes in CKD IRIS stages 2 and 3. A study investigating renal cortical thickness using ultrasonography in patients with CKD found a significant difference in cortical thickness between IRIS stages 2 and 3 (5). Minimal changes in cortical thickness occur in the early disease stages, with a marked decrease in the later stages due to compensatory hypertrophy of the outer medulla, which is typically considered the renal cortex. Furthermore, a study observed a significant increase in the number of dogs with three or more abnormal ultrasound findings as they progressed from CKD IRIS stages 2–4 (4). There have also been reports of significant differences in prognosis between IRIS stages 2 and 3 (29). According to other research, compared with dogs diagnosed with IRIS stages 1 and 2, dogs diagnosed with IRIS stage 3 had a 2.62-fold higher risk of CKD-related death (95% confidence interval [CI]: 1.14–6.01, *p* = 0.023), and dogs diagnosed with IRIS stage 4 showed a 4.71-fold higher risk (95% CI: 1.74–12.72, *p* = 0.002) (3).

In the task of discriminating IRIS stage 3 or higher, which exhibited the highest performance in the binary classification, the performance of AI surpassed that of radiologists. Although kidney ultrasonography is a routine and straightforward task, the accuracy of radiologists is presumed to be low because they typically do not consider the prediction of IRIS stages when conducting renal ultrasonographic examinations. In contrast, the AI model that was specifically trained for this classification task demonstrated excellent performance. Through comparison experiments with radiologists, it was confirmed that the AI model could be an effective substitute for experts.

The intra-observer reliability among the individual radiologists reached a significant level, indicating consistent diagnostic criteria for each radiologist. However, there was a moderate level of variability between different radiologists (inter-observer variability), suggesting divergent diagnostic criteria among them. In this context, deep learning-assisted ultrasound diagnosis can improve diagnostic objectivity by providing consistent criteria and capabilities. Moreover, by serving as a virtual discussion partner for radiologists, it can aid in decision-making processes.

This study had some limitations. First, as a single-center study conducted using only two types of ultrasound devices, the dataset primarily comprised images generated from a single ultrasound machine owing to the recent introduction of a new device at this center. Consequently, there is a risk of the AI model becoming overly adapted to images from specific equipment, potentially hindering its ability to generalize. Second, compared with similar studies in human medicine, the present animal study had a smaller sample size and dataset, which can increase the risk of overfitting in AI models owing to the lack of data diversity. Therefore, efforts were made throughout the experimental design to prevent overfitting, as aforementioned. Hence, further research using larger multicenter datasets is warranted. Alternatively, innovative approaches, such as generative models, may offer potential solutions to address the ongoing challenge of limited data in veterinary AI research. Finally, this study did not directly incorporate the glomerular filtration rate (GFR) as a measure of renal function. Expanding quantitative prediction studies to include variables, such as GFR or SDMA, could provide valuable insights into renal function assessment.

In conclusion, this study developed a deep learning framework capable of reliably classifying CKD IRIS stages 3 and 4 in dogs using ultrasonograms. The developed framework demonstrated higher accuracy than veterinary imaging specialists and provided more objective and consistent interpretations. Hence, deep-learning-based ultrasound diagnostics have been proven to be potentially valuable tools for diagnosing CKD in dogs.

## Data availability statement

The original contributions presented in the study are included in the article/Supplementary material, further inquiries can be directed to the corresponding author.

## Author contributions

HY: Conceptualization, Writing – original draft, Data curation, Formal analysis, Investigation. I-GL: Writing – original draft, Methodology, Software. J-YO: Methodology, Software, Writing – review & editing. JK: Writing – review & editing. J-HJ: Supervision, Writing – review & editing. KE: Supervision, Writing – review & editing.

## References

[1] Bartges JW. Chronic kidney disease in dogs and cats. Vet Clin North Am Small Anim Pract. (2012) 42:669–92. 10.1016/j.cvsm.2012.04.008 22720808

[2] Polzin DJ. Chronic kidney disease in small animals. Vet Clin North Am Small Anim Pract. (2011) 41:15–30. 10.1016/j.cvsm.2010.09.004 21251509

[3] O'Neill DG Elliott J Church DB McGreevy PD Thomson PC Brodbelt DC. Chronic kidney disease in dogs in UK veterinary practices: prevalence, risk factors, and survival. J Vet Intern Med. (2013) 27:814–21. 10.1111/jvim.12090 23647231

[4] Perondi F Lippi I Marchetti V Bruno B Borrelli A Citi S. How ultrasound can be useful for staging chronic kidney disease in dogs: ultrasound findings in 855 cases. Vet Sci. (2020) 7:147. 10.3390/vetsci7040147 33019496 PMC7712280

[5] Choo D Kim SS Kwon D Lee K Yoon H. Ultrasonographic quantitative evaluation of acute and chronic renal disease using the renal cortical thickness to aorta ratio in dogs. Vet Radiol Ultrasound. (2023) 64:140–8. 10.1111/vru.13154 36049077

[6] Siddappa JK Singla S Al Ameen M Rakshith SC Kumar N. Correlation of ultrasonographic parameters with serum creatinine in chronic kidney disease. J Clin Imaging Sci. (2013) 3:28. 10.4103/2156-7514.114809 24083065 PMC3779384

[7] Chartrand G Cheng PM Vorontsov E Drozdzal M Turcotte S Pal CJ. Deep learning: a primer for radiologists. Radiographics. (2017) 37:2113–31. 10.1148/rg.2017170077 29131760

[8] Jeong J-H Shim K-H Kim D-J Lee S-W. Brain-controlled robotic arm system based on multi-directional CNN-BiLSTM network using EEG signals. IEEE Trans Neural Syst Rehabil Eng. (2020) 28:1226–38. 10.1109/TNSRE.2020.2981659 32191894

[9] Jeong J-H Lee I-G Kim S-K Kam T-E Lee S-W Lee E. DeepHealthNet: adolescent obesity prediction system based on a deep learning framework. IEEE J Biomed Health Inform. (2024) 28:2282–93. 10.1109/JBHI.2024.3356580 38315595

[10] Ronneberger O Fischer P Brox T. U-net: Convolutional networks for biomedical image segmentation. In: Medical Image Computing and Computer-Assisted Intervention–MICCAI 2015: 18th International Conference, Munich, Germany, October 5-9, 2015, Proceedings, Part III 18. Cham: Springer International Publishing (2015). p. 234–241.

[11] Liang X Du M Chen Z. Artificial intelligence-aided ultrasound in renal diseases: a systematic review. Quant Imaging Med Surg. (2023) 13:3988–4001. 10.21037/qims-22-1428 37284081 PMC10240007

[12] Hao P Xu Z Tian S Wu F Chen W Wu J. Texture branch network for chronic kidney disease screening based on ultrasound images. Front Inform Technol Electron Eng. (2020) 21:1161–70. 10.1631/FITEE.1900210

[13] Zhang L Chen Z Feng L Guo L Liu D Hai J. Preliminary study on the application of renal ultrasonography radiomics in the classification of glomerulopathy. BMC Med Imaging. (2021) 21:1. 10.1186/s12880-021-00647-8 34301205 PMC8305820

[14] Kuo CC Chang CM Liu KT Lin WK Chiang HY Chung CW. Automation of the kidney function prediction and classification through ultrasound-based kidney imaging using deep learning. NPJ Digit Med. (2019) 2:29. 10.1038/s41746-019-0104-2 31304376 PMC6550224

[15] Gomes DA Alves-Pimenta MS Ginja M FilipeV. Predicting canine hip dysplasia in x-ray images using deep learning. In: International Conference on Optimization, Learning Algorithms and Applications. Cham: Springer International Publishing (2021). p.393–400. 10.1007/978-3-030-91885-9_29

[16] Li S Wang Z Visser LC Wisner ER Cheng H. Pilot study: application of artificial intelligence for detecting left atrial enlargement on canine thoracic radiographs. Vet Radiol Ultrasound. (2020) 61:611–8. 10.1111/vru.12901 32783354 PMC7689842

[17] Banzato T Wodzinski M Tauceri F Donà C Scavazza F Müller H. An AI-based algorithm for the automatic classification of thoracic radiographs in cats. Front Vet Sci. (2021) 8:731936. 10.3389/fvets.2021.731936 34722699 PMC8554083

[18] Banzato T Bernardini M Cherubini GB Zotti A. Texture analysis of magnetic resonance images to predict histologic grade of meningiomas in dogs. Am J Vet Res. (2017) 78:1156–62. 10.2460/ajvr.78.10.1156 28945125

[19] Ji Y Cho H Seon S Lee K Yoon H. A deep learning model for CT-based kidney volume determination in dogs and normal reference definition. Front Vet Sci. (2022) 9:1011804. 10.3389/fvets.2022.1011804 36387402 PMC9649823

[20] Ji Y Hwang G Lee SJ Lee K Yoon H A. deep learning model for automated kidney calculi detection on non-contrast computed tomography scans in dogs. Front Vet Sci. (2023) 10:1236579. 10.3389/fvets.2023.1236579 37799401 PMC10548669

[21] Jocher G Chaurasia A Qiu J. Ultralytics YOLO (Version 8.0.0) [Computer Software]. (2023). Available online at: https://github.com/ultralytics/ultralytics

[22] Akula SP Akula P Kamati N. Detection and classification of canine hip dysplasia according to FCI grading system using 3D CNN's. In: 2022 First International Conference on Artificial Intelligence Trends and Pattern Recognition (ICAITPR). Hyderabad: IEEE (2022). p. 1–6. 10.1109/ICAITPR51569.2022.9844209

[23] Kulkarni A Chong D Batarseh FA. Foundations of data imbalance and solutions for a data democracy. In: Data Democracy. Cambridge, MA: Academic Press (2020). p.83–106. 10.1016/B978-0-12-818366-3.00005-8

[24] Banzato T Wodzinski M Burti S Osti VL Rossoni V Atzori M. Automatic classification of canine thoracic radiographs using deep learning. Sci Rep. (2021) 11:3964. 10.1038/s41598-021-83515-3 33597566 PMC7889925

[25] Hennessey E DiFazio M Hennessey R Cassel N. Artificial intelligence in veterinary diagnostic imaging: a literature review. Vet Radiol Ultrasound. (2022) 63:851–70. 10.1111/vru.13163 36468206

[26] Astivia OLO Zumbo BD. Population models and simulation methods: the case of the Spearman rank correlation. Br J Math Stat Psychol. (2017) 70:347–67. 10.1111/bmsp.12085 28140458

[27] Landis JR Koch GG. The measurement of observer agreement for categorical data. Biometrics. (1977) 33:159–74. 10.2307/2529310 843571

[28] Odeh R Noone D Bowlin PR Braga LH Lorenzo AJ. Predicting risk of chronic kidney disease in infants and young children at diagnosis of posterior urethral valves: initial ultrasound kidney characteristics and validation of parenchymal area as forecasters of renal reserve. J Urol. (2016) 196:862–68. 10.1016/j.juro.2016.03.137 27017936

[29] Rudinsky AJ Harjes LM Byron J Chew DJ Toribio RE Langston C. Factors associated with survival in dogs with chronic kidney disease. J Vet Intern Med. (2018) 32:1977–82. 10.1111/jvim.15322 30325060 PMC6271312

