# Supplementary material for: Deep learning-based ultrasonographic classification of canine chronic kidney disease
Source: Front Vet Sci. 2024 Sep 4;11:1443234. doi: 10.3389/fvets.2024.1443234 (PMC11408351; doi:10.3389/fvets.2024.1443234)
Supplement: Supplementary file 1 [file Data_Sheet_1.docx]

Supplementary Material

**Supplementary Figure 1**. Confusion matrices of the developed models

Panel (A) depicts the multiclass classification model, while Panels (B) through (E) depict cases 1 through 4 of the binary classification model. The label 'Normal' refers to the lower stage of chronic kidney disease according to the International Renal Interest Society stage in binary classification, while the label 'Abnormal' indicates the higher stage..


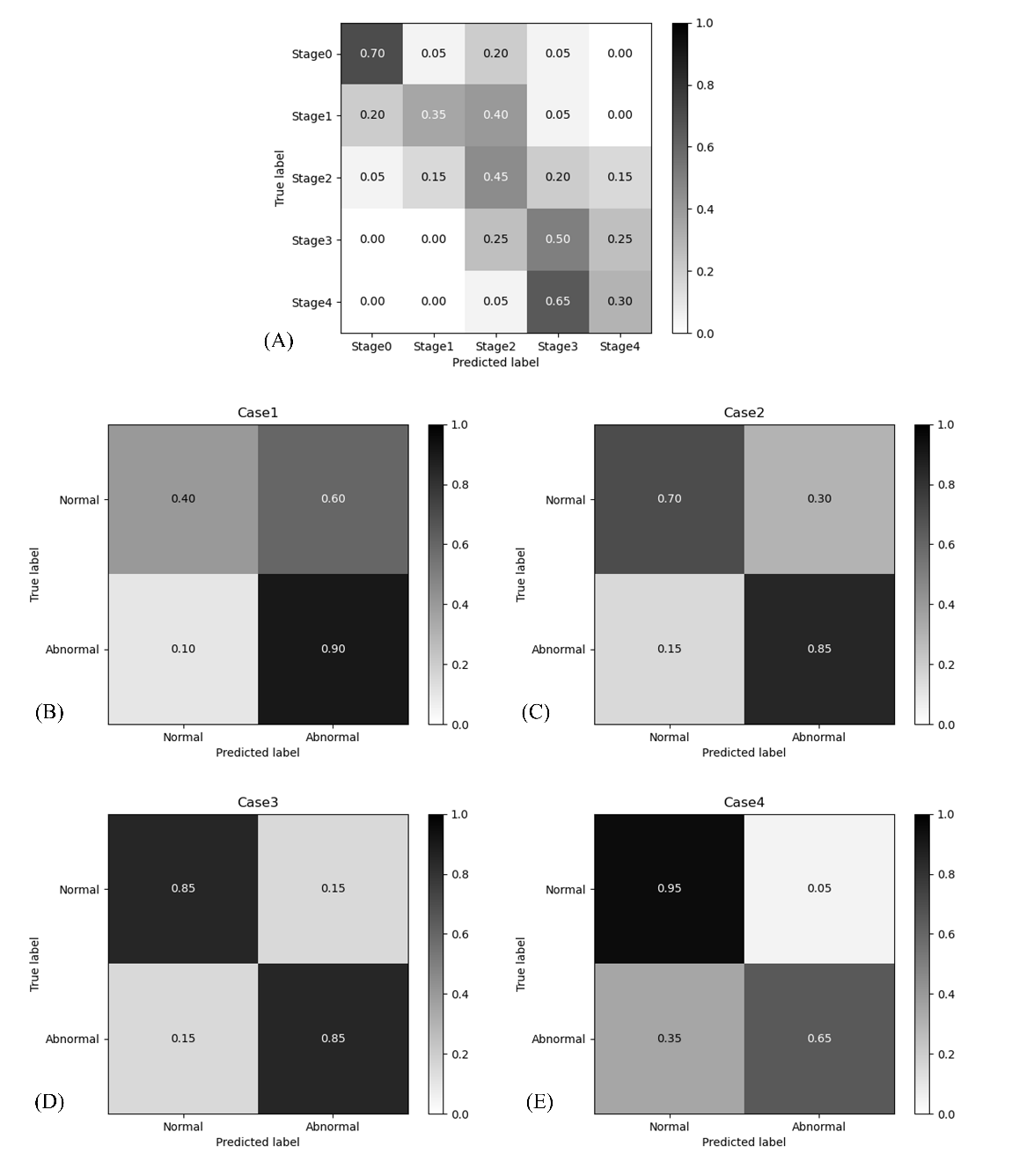


**Supplementary Table 1.** Demographics and clinical data of the study population

|  | | Total  (n = 198) | | | | | | Stage 0  (n = 39) | Stage 1  (n = 44) | Stage 2  (n = 51) | Stage 3  (n = 35) | Stage 4  (n = 29) |
| --- | --- | --- | --- | --- | --- | --- | --- | --- | --- | --- | --- | --- |
| Age (years)^a^ | | | | 11.0 (8.0–14.0) | | | | 4.0 (2.5–6.0) | 11.0 (10.0–13.0) | 13.0 (5.9–7.2) | 13.0 (8.50–14.0) | 14.0 (11.0–16.0) |
| Sex (n, %)^b^ | | | | | |  | |  |  |  |  |  |
| Male | Intact | | | | 7, 3.5% | | 1, 2.5% | | 3, 6.8% | 1, 1.9% | 2, 5.7% | 0, 0% |
|  | Castrated | | | | 104, 52.5% | | 24, 61.5% | | 20, 45.4% | 28, 54.9% | 16, 45.7% | 16, 55.1% |
| Female | Intact | | | | 7, 3.5% | | 2, 5.1% | | 1, 2.2% | 0, 0% | 2, 5.7% | 2, 6.8% |
|  | Spayed | | | | 80, 40.4% | | 12, 30.7% | | 20, 45.4% | 22, 43.1% | 15, 42.8% | 11, 37.9% |
| Clinical data ^a^ | | |  | | | | |  |  |  |  |  |
| LK/AO ^c^ ratio | | | 6.7 (5.9–7.5) | | | | | 6.9 (6.2–7.6) | 7.1 (5.8–7.8) | 6.7 (5.9–7.2) | 6.5 (6.0–7.7) | 6.5 (5.8–7.7) |
| RK/AO ^d^ ratio | | | 7.1 (6.1–7.7) | | | | | 7.0 (6.4–7.6) | 6.9 (6.3–7.6) | 7.1 (5.7–7.8) | 7.3 (6.2–8.3) | 7.1 (5.8–8.2) |
| SDMA ^e^ (µg/dL) | | | 20.0 (13.0–36.2) | | | | | 9.0 (7.0–11.0) | 14.0 (12.0–15.0) | 22.0 (19.0–27.0) | 38.0 (30.2–41.7) | 74.5 (53.2–78.2) |
| Creatinine (mg/dL) | | | | | 1.2 (0.8–2.0) | | | 0.8 (0.7–1.1) | 0.95 (0.7–1.1) | 1.3 (0.9–1.4) | 2.0 (1.5–2.9) | 5.3 (3.4–6.1) |
| Urea (mg/dL) | | | 28.0 (17.2–51.0) | | | | | 16.0 (12.0–20.0) | 0.95 (0.7–1.1) | 29.0 (20.5–32.5) | 54.0 (30.5–73.0) | 104.0 (39.0–185.0) |
| UPC ^f^ ratio | | | 1.0 (0.3–2.4) | | | | | 0.2 (0.06–0.4) | 0.8 (0.3–3.6) | 0.6 (0.1–4.5) | 1.4 (1.0–2.4) | 1.8 (0.9–2.6) |
|  | | |  | | | | |  |  |  |  |  |

^a^, data was presented as median with interquartile range (IQR).

^b^, data was presented as the number of the dogs (n) and percentage (%)

^c^, ultrasonographic left kidney length to abdominal aortic diameter ratio

^d^, ultrasonographic right kidney length to abdominal aortic diameter ratio

^e^, blood symmetric dimethyl arginine

^f^, urine protein to creatinine ratio

**Supplementary Table 2.** Interobserver agreement among radiologists

|  | Kappa value* | SE | 95% CI | p-value** |
| --- | --- | --- | --- | --- |
| All radiologist | 0.602 | 0.0316 | 0.241 – 0.962 | < .001 |

SE: standard error

CI: confidence interval

*, a value of 1 indicates perfect agreement

**, p-value < 0.05 was considered statistically significant in Cohen's (unweighted) kappa coefficient

**Supplementary Table 3.** Intraobserver reliability among radiologists

| Radiologist | Agreement between the  two assessments | | Kappa value* | p-value** |
| --- | --- | --- | --- | --- |
|  | Agree (n/60) | Disagree (n/60) |  |  |
| 1 | 54 | 6 | 0.797 | < .001 |
| 2 | 49 | 11 | 0.667 | < .001 |
| 3 | 57 | 3 | 0.879 | < .001 |
| 4 | 56 | 4 | 0.855 | < .001 |

*, a value of 1 indicates perfect agreement

**, p-value < 0.05 was considered statistically significant in Cohen's (unweighted) kappa coefficient
